# Learning styles and learning preferences of higher diploma students

**DOI:** 10.1186/2193-1801-3-S1-O9

**Published:** 2014-12-04

**Authors:** Janet, Man-wai Cheung, Joanna, Kar-wai Cheng, Alan, Nga-lun Wong

**Affiliations:** Centre for Learning and Teaching, Vocational Training Council, Kragujevac, Hong Kong

## Background

The student diversity in the Vocational Training Council (VTC) has been increasing in recent years. With most students coming from the Post 90s Generation, they share characteristics of the Net Generation, who are ‘associated with new forms of sociality’[1]. It is therefore important to update teachers to the learning styles and learning preferences of their students. Learning style is defined as ‘the manner in which an individual perceives and processes information in learning situations’, and the VARK model is adopted to analyze learning style. Learning preference is ‘the choice of one learning situation or condition over another’[2] and concerns students’ choices in learning such as preferred mobile learning activities, learning tools, communication media and video clips. To collect updated data for reference, this research aims to study whether there is any significant difference among Higher Diploma students of the seven disciplines in terms of learning styles and learning preferences.

## Methods

Data were collected via online survey on learning habits and unstructured interviews with students. The online survey was open for all full-time students from February to April 2014, with over 4000 responses. For VARK learning style, paper questionnaires were distributed to students via Discipline Planning Offices from which 2514 responses were collected. Interviews with students of the seven disciplines were conducted at different campuses in May 2014.

## Results

The data reflected that the students shared some demographic commonalities: most of the students were 18-20 years old; about 70% graduated from Secondary Six of the New Senior Secondary Academic Structure; over 90% claimed that their most fluent language was Chinese/Cantonese in writing/speaking; each of them was equipped with 1.82 mobile devices on average; over 60% used Android as the operating system of their mobile devices; and about 80% of the students use a 3G/4G mobile network, among which 71% used an unlimited data plan.

Search engines, video search sites and WhatsApp were the most popular communication media among the disciplines, with 89%, 65% and 59% popularity recorded, respectively. Frequent use of Facebook (70%) was reported for communication, but it was not equally important in learning (39%).

Yet some differences among disciplines were found: use of forums in learning was popular in the Engineering and Information Technology disciplines only; email was important for Business Administration, Design and Hotel and Service & Tourism Studies, but it was not so important for students in other disciplines.

Students’ learning styles were analyzed with Fleming’s VARK model[3], the acronym of which stands for ‘visual’, ‘aural’, ‘read/write’ and ‘kinesthetic’. The combinations of the VARK are classified as ‘single preference’, ‘bimodal’, ‘trimodal’, ‘multi-modal type one’, ‘multi-modal type two’ and ‘multi-modal transition’. A chi-square test was performed to investigate whether convincing evidence could be found in differences of VARK profiles among the seven disciplines. Significant differences in the distribution of VARK profiles of students among the disciplines were found: X² (84, N = 2514 ) = 130.6, p = 0.001 (< 0.05]. Significant contributors to the differences were VA (BA), VA and VK (CECS), VK (DE), K (ENG), VK (HoSTs) and K(IT).

In the interviews, the students reported their habit of using social media to aid in learning, such as in the discussion of projects, file sharing, dictionary use and information searches. Interactive lessons such as tutorials, workshops and laboratory exercises were more welcome than one-way dissemination of knowledge.

## Conclusions

Teaching staff could enrich their teaching materials and strategies in view of the popularity of mobile devices, immersion in the digital life and the diverse learning habits among students. It is possible to explore various discipline-specific teaching tools and strategies because differences in data among the disciplines were found.
